# Ecological genetics of isolated loach populations indicate compromised adaptive potential

**DOI:** 10.1038/s41437-024-00695-0

**Published:** 2024-07-03

**Authors:** Xi Wang, Kerry Reid, Ying Chen, David Dudgeon, Juha Merilä

**Affiliations:** 1 Area of Ecology and Biodiversity, School of Biological Sciences, The University of Hong Kong, Hong Kong SAR, China; 2 Ecological Genetics Research Unit, Organismal and Evolutionary Biology Programme, University of Helsinki, FI-00014 University of Helsinki, Helsinki, Finland

**Keywords:** Population genetics, Population dynamics, Ecological genetics

## Abstract

Many endangered species live in fragmented and isolated populations with low genetic variability, signs of inbreeding, and small effective population sizes - all features elevating their extinction risk. The flat-headed loach (*Oreonectes platycephalus)*, a small noemacheilid fish, is widely across southern China, but only in the headwaters of hillstreams; as a result, they are spatially isolated from conspecific populations. We surveyed single nucleotide polymorphisms in 16 Hong Kong populations of *O. platycephalus* to determine whether loach populations from different streams were genetically isolated from each other, showed low levels of genetic diversity, signs of inbreeding, and had small contemporary effective population sizes. Estimates of average observed heterozygosity (*H*_*O*_ = 0.0473), average weighted nucleotide diversity (*π*_*w*_ = 0.0546) and contemporary effective population sizes (*N*_*e*_ = 10.2 ~ 129.8) were very low, and several populations showed clear signs of inbreeding as judged from relatedness estimates. The degree of genetic differentiation among populations was very high (average *F*_*ST*_ = 0.668), even over short geographic distances (<1.5 km), with clear patterns of isolation by distance. These results suggest that Hong Kong populations of *O. platycephalus* have experienced strong genetic drift and loss of genetic variability because sea-level rise after the last glaciation reduced connectedness among paleodrainages, isolating populations in headwaters. All this, together with the fact that the levels of genetic diversity and contemporary effective population sizes within *O. platycephalus* populations are lower than most other freshwater fishes, suggests that they face high local extinction risk and have limited capacity for future adaptation.

## Introduction

Changing climatic conditions pose severe challenges for local populations of many organisms and threaten their existence (Albano et al. 2021). Species with high dispersal ability can shift their ranges to include more thermally suitable habitats, leading to the redistribution of global biodiversity (Pecl et al. 2017). For instance, it has been predicted that, by 2070, the climatic niches of around 30% of birds and mammals will include countries where they have never lived previously (Titley et al. 2021). However, migration to new areas is not an option for many non-vagile species in isolated habitat patches, such as lakes and mountaintops. Phenotypic plasticity may provide means for them to acclimate to warmer temperatures (Gienapp et al. 2008) but it is unlikely that such responses will allow populations to keep pace with rising temperatures indefinitely (Cerini et al. 2023). Furthermore, evolutionary adaptation to new environmental conditions is possible only if there is sufficient genetic variation in the population to allow response to natural selection (Lynch and Walsh 1998).

Genetic diversity is the fuel that natural selection needs to allow organismal adaptation to changing environmental conditions. Whether adaptation to changing environmental conditions will occur depends critically on populations’ access to genetic variation in the traits influencing fitness. The likelihood of adaptation is the function of effective population size: larger populations harbor more genetic variation than smaller ones, and natural selection is more efficient in the former (Lanfear et al. 2014; Saccheri and Hanski 2006). However, large populations are also larger targets for new mutations and are therefore expected to be burdened with relatively high loads of deleterious recessive mutations (Grossen et al. 2020; Saccheri and Hanski 2006). Furthermore, once the size of formerly large populations becomes reduced – for instance due to climate change-imposed fitness loss – there is a risk that segregating deleterious mutations will become expressed through inbreeding causing significant maladaptation (Fraimout et al. 2023; Grossen et al. 2020; Robinson et al. 2019).

**Fig. 1:**
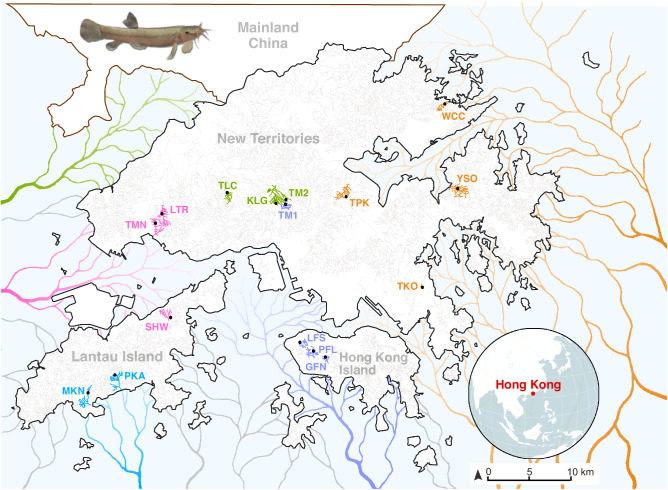
Study populations of flat-headed loaches. Map of the study area showing the sampled hillstreams in the territory of Hong Kong with different palaeo-drainages indicated in color (green: Tai Mo Shan drainage - TMS; pink: West New Territories drainage - NTW; blue: South Lantau drainage - LTS; purple: Hong Kong Island drainage - HKI; orange: East New Territories drainage - NTE; gray: Other paleodrainages not included in this study) redrawn from Fyfe et al. (2000). Light blue depicts the current marine area. Fish insert at the top (left) depicts the study species, the flat-headed loach. For locality abbreviations, see Supplementary Table S1 (Photo courtesy: Chi Kit Yeung).

The flat-headed loach (*Oreonectes platycephalus*: Noemacheilidae; Fig. 1) is a small freshwater fish that lives in the headwaters of hillstreams in Hong Kong and southern China (Du et al. 2008; Dudgeon 2003). The species’ wide distribution and confinement to the top parts of hillstreams suggest that it colonized its current habitats post-glacially. Since then, many previously connected streams have become isolated from each other due to ca. 150 m sea level rise after the final glacial maximum at 12,000 years BP (Fyfe et al. 2000). As a result, most populations of this loach in Hong Kong are isolated from each other and hence can be expected to have been subject to attrition of genetic diversity due to genetic drift and inbreeding. Furthermore, given that post-glacially formed habitats of coastal southeast China are young (Fyfe et al. 1999), there has been little time to restore any lost genetic variation through mutations, because mutation rates in fish are low (Bergeron et al. 2023; Zhang et al. 2023).

Many freshwater systems can be considered to be metapopulations whose evolutionary and genetic dynamics are strongly influenced by river or lake network complexity, as well as by steep physical gradients over short geographic distances (Labonne et al. 2008; Paz‐Vinas and Blanchet 2015). The dendritic connectivity of Hong Kong hillstreams is low, and many hillstreams are short, discharging directly to the sea, meaning that the loaches and other freshwater species in different hillstreams are effectively isolated from each other (Tsang et al. 2016; Wong et al. 2017). This isolation traces back to the Pleistocene when the sea levels were 150 m lower and the exposed landmass that extended over 120 km south from the modern coastline (Fyfe et al. 2000). During this time, the hillstreams did not discharge directly to the sea, but joined to several paleodrainages (Fig. 1) allowing connectivity between now isolated loach populations. After these connections were lost, loach populations in different streams would have evolved independently without opportunities for evolutionary or genetic rescue through gene flow (Bell 2017; Whiteley et al. 2015).

This study represents the first step towards understanding the history, population structure, and evolutionary potential of *O. platycephalus* by conducting a population genomic survey of genetic diversity and differentiation across the territory of Hong Kong. Given what little is known about the biology of this species (mostly its feeding behavior; Dudgeon 1991; 1993), we expected to find a strong population structure attributable to strong genetic drift and lack of gene flow among drainages. We further expected to find low levels of genetic diversity, signs of inbreeding, and hence, reduced adaptive potential in the face of changing environmental conditions. We also assessed the phylogeographic history of the study populations with the aid of the reconstructed geological history of drainage systems in Hong Kong, as well as their contemporary effective population sizes.

## Materials and methods

### Sample collection and DNA extractions

A total of 282 flat-headed loaches were collected from 16 sites in Hong Kong: four sites from East New Territories, six sites from West New Territories, three sites on Hong Kong Island and three sites on Lantau Island, with an average sample size of 18 individuals per population (n = 5–20; Fig. 1, Tables 1 and Supplementary Table S1). The sampling was conducted in January–March 2022 with dip-nets at night when flat-headed loaches are most active. All specimens were euthanized with MS-222 in the field and stored in 95% ethanol maintained at room temperature until genomic DNA was extracted from the pectoral fins using the *DNeasy Blood and Tissue Kit* (Qiagen, Germany) following the manufacturer’s recommendations. The DNA concentration for each sample was assessed by *NanoDrop One spectrophotometer* (Thermo Fisher, US) and diluted to 50 ng/μL for reduced representation library preparations and sequencing.

**Table 1:** Estimates of genetic diversity of the 16 flat-headed loach populations.

Population	Paleodrainage system	*N*_*IND*_	*H*_*O*_	*H*_*E*_	*Ar*	*π*_*w*_	*F*_*IS*_	*F*_*IS*_ 95%CI Low	*F*_*IS*_ 95%CI High
YSO	NTE	5	0.0344	0.0425	1.0398	0.0368	0.1906^a^	0.1170	0.2057
TKO	NTE	18	0.0558	0.0628	1.0618	0.0595	0.1115^a^	0.0785	0.1294
TPK	NTE	19	0.0381	0.0446	1.0436	0.0416	0.1457^a^	0.1058	0.1654
WCC	NTE	19	0.0392	0.0430	1.0423	0.0396	0.0884^a^	0.0542	0.1037
PKA	LTS	19	0.0345	0.0380	1.0375	0.0361	0.0921^a^	0.0524	0.1094
MKN	LTS	19	0.0334	0.0349	1.0345	0.0331	0.0430^a^	0.0061	0.0633
SHW	NTW	15	0.0019	0.0022	1.0020	0.0018	0.1364	−0.1572	0.3088
LTR	NTW	19	0.0627	0.0763	1.0752	0.0745	0.1782^a^	0.1536	0.1917
TMN	NTW	20	0.0804	0.1072	1.1060	0.1061	0.2500^a^	0.2314	0.2658
TLC	TMS	19	0.0656	0.0859	1.0849	0.0846	0.2363^a^	0.2156	0.2551
KLG	TMS	19	0.0521	0.0618	1.0609	0.0600	0.1570^a^	0.1293	0.1753
TM2	TMS	18	0.0626	0.0782	1.0770	0.0767	0.1995^a^	0.1743	0.2169
TM1	HKI	19	0.0469	0.0602	1.0584	0.0579	0.2209^a^	0.1858	0.2386
LFS	HKI	19	0.0536	0.0613	1.0604	0.0591	0.1256^a^	0.0975	0.1418
GFN	HKI	18	0.0435	0.0483	1.0472	0.0464	0.0994^a^	0.0641	0.1234
PFL	HKI	17	0.0519	0.0616	1.0608	0.0603	0.1575^a^	0.1318	0.1764

*N*_*IND*_ Number of individuals, *H*_*O*_ Observed Heterozygosity, *H*_*E*_ Expected Heterozygosity, *Ar* Allelic richness, *π*_*w*_ Average weighted nucleotide diversity, *F*_*IS*_ Inbreeding Coefficient, estimated as *F*_*IS*_ = 1 − (*H*_*O*_/*H*_*E*_).

^a^Indicates statistically significant inbreeding.

### DarT sequencing

The 282 loach DNA samples were sequenced at *Diversity Arrays Technology Pty Ltd, Australia* with DArTseq™ (Diversity Arrays Technology Sequencing Technology) for medium-density sequencing which combines genome complexity reduction methods with next-generation sequencing (Kilian et al. 2012). Libraries were constructed using the DArTseq™ complexity reduction method described here in brief: (i) genomic DNA was digested with two restriction enzymes (*PstI* and *MseI)*; (ii) DNA fragments were ligated to the barcoded adapters; after which (iii) adapter-ligated fragments were amplified with PCR. DNA libraries were sequenced using Single-Read sequencing runs for 77 cycles (Egea et al. 2017). High-throughput sequencing was implemented with the Illumina HiSeq2500 machine (Illumina, USA) and DArTseq™ marker scoring was performed using DArTsoft (Kilian et al. 2012). Single-nucleotide polymorphisms (SNPs) were scored as “0” = reference allele homozygote, “1” = alternative allele homozygote and “2” = heterozygote (Uba et al. 2021).

### SNP filtering

SNPs called from DArTseq™ were analyzed in *R v4.2.1* using package *dartR v2.7.2* (Gruber et al. 2018; Mijangos et al. 2022) which was developed to access and explore SNP data obtained with DArTseq™. Approximately 20 Gb of DArTseq data (466.78 million raw reads) were generated and calling of sequence variants generated a total of 56,416 SNPs.

The following filtering criteria were applied to exclude SNPs in the given order (steps “i” to “vi” are default settings for SNPs quality controls; details of filtering setting can be found in Supplemental Material): (i) reproducibility <0.99; (ii) coverage <5X or >50X (coverage automatically calculated by dartR); (iii) SNPs with sequence tag length <20 or >69; (iv) SNP position outside the trimmed sequence tag; (v) secondary SNPs; (vi) pairwise Hamming distance between sequence tags <0.2; (vii) call rate <0.7; (viii) minor allele count (MAC) < 3. After these filtering steps, 7045 SNPs were kept for downstream analyses.

### Genetic diversity and population dynamics

The observed heterozygosity (*H*_*O*_), expected heterozygosity (*H*_*E*_), allelic richness (*Ar*), inbreeding coefficient (*F*_*IS*_) and 95% CI for *F*_*IS*_ (1000 bootstrap replicates) for each population were calculated with R package *hierfstat v0.5-11* (Goudet 2005). The nucleotide diversity (*π*) for each locus in each population was estimated by *vcftools v0.1.17* (--site-pi) (Danecek et al. 2011). The average weighted nucleotide diversity (*π*_*w*_) of each population was calculated following Konopiński (2023) as:

1 $${\pi }_{w}=\frac{{\Sigma }^{{N}_{loci}}\pi }{N}$$

where *N*_*loci*_ is the number of loci that do not have all missing data in a population; *N* is the number of SNPs in this study (*N* = 7045).

A relatedness statistic of individuals within each population was also calculated by *vcftools v0.1.17* based on the KING (Kinship-based INference for Genome-wide association studies) inference (Manichaikul et al. 2010) and the results were plotted by R package *pheatmap v1.0.12* (Kolde 2019). According to the KING tutorial, an estimated kinship coefficient range >0.354, (0.177, 0.354], (0.088, 0.177] and (0.044, 0.088] corresponds to duplicate/twin, first-degree, second-degree, and third-degree relationships, respectively (Manichaikul et al. 2010). Analysis of variance (ANOVA) of relatedness among the 16 populations was conducted in *R* with *p*-value adjusted using the FDR method with the package *agricolae v1.3-7* (Mendiburu 2019). The contemporary effective population size (*N*_*e*_) of each population was estimated by *NeEstimator v2.1* based on the Linkage Disequilibrium method with singletons removed (Do et al. 2014). Linear mix-models assessing the effects of geographical factors (altitude, summed length of streams) and relatedness of individuals within each population on *π* and *N*_*e*_, were estimated with *R package MCMCglmm v.2.35* (Hadfield 2010) with phylogenetic correlation matrix (converted from neighbor‐joining tree generated by dartR with default setting; NJ) as a random effect (model: list(G = list(G1 = list(V = 1, nu = 1, alpha.mu = 0, alpha.V = 1000)), R = list(V = 1, nu = 0.002)); nitt = 1001000, thin = 1000, burnin = 10000). The demographic history of each population was estimated with Stairway Plot v2.1.1 (Liu and Fu 2020), based on the folded site-frequency spectrum (SFS) calculated using *dartR*, with a mutation rate of 0.562e-8 mutations/site/generation (estimated for common carp reported by Bergeron et al. 2023) and a generation time of two years. In the existing fish mutation rate studies (Zhang et al. 2023), common carp is the species with the closest phylogenetic relationship to *O. platycephalus*. Since mutation rates in fish are not highly variable (Zhang et al. 2023) it is reasonable to assume that the common carp estimate is a good proxy for *O. platycephalus* mutation rate. As the generation time of *O. platycephalus* is unknown, inferred generation time of *Nemacheilus triangularis* (2.2 years; https://fishbase.mnhn.fr/summary/Nemacheilus-triangularis), a closely related species from the same subfamily (Nemacheilidae), was used.

### Population structure

Pairwise *F*_*ST*_ values between populations were estimated with *dartR* based on the implementation in the *StAMPP package* (Pembleton et al. 2013) with 1000 bootstrap replicates and a heatmap showing pairwise *F*_*ST*_ results was plotted with an R package *pheatmap*. To see whether levels of differentiation as measured by *F*_*ST*_ differed within and among each paleodrainage, we compared mean *F*_*ST*_ values using *t*-tests. The neighbor‐joining (NJ) tree was constructed based on *F*_*ST*_ values obtained with *dartR* using default settings. Population structure was also assessed using a maximum likelihood estimation of individual ancestries by *ADMIXTURE v1.3.0* (Alexander et al. 2009). Assuming K ranging from 1 to 20, cross-validation information of each K was collected and the K with the lowest cross-validation error was chosen to plot population ancestries. Principal Component Analysis (PCA) was conducted by *dartR* (nfactors = 2) to assess population structure. A map of loach sampling localities with PCA results was drawn by *Adobe Illustrator 2022* (Adobe Inc., US). Isolation by distance (IBD) analyses (geographic distance calculated by coordinates and by shortest possible waterway) based on the Mantel test were performed with *dartR* using pairwise *F*_*ST*_ values with Rousset’s correction (Rousset 1997) as genetic distances. The shortest possible waterway between each population pair was estimated based on the map of paleodrainage systems (Fig. 1). If the populations were within the same drainage, the length of the stream connecting the two populations was measured. If the populations were in different drainage systems, the shortest waterway through the sea was measured as a proxy of “paleodistance”. The latter measure was adopted as paleodrainage reconstructions were not possible for all drainages (and between drainages) because reconstructions did not extend to cover all relevant areas (cf. Fig. 1).

To compare the level of genetic differentiation among loach populations to that of other freshwater fishes, we compiled data from recent (2013–2023; see Supplementary Table S6 and Supplementary Material) studies which have estimated *F*_*ST*_ among freshwater fish populations using reduced-representation genome sequencing approaches. We found a total of 27 studies (See Supplementary Table S7 and Supplementary Material for details) and extracted the mean pairwise *F*_*ST*_ estimate from each of them. We further estimated the geographic coverage in each of these studies by measuring the area (in km^2^) their sampling covered (i.e. the area contained within the perimeter drawn around furthest sampling localities). Plotting the mean *F*_*ST*_ from each study as a function of the geographic extent of sampling allowed us to explore how the loach *F*_*ST*_ value compared to other fishes. Linear models assessing *F*_*ST*_ as a function of (log) geographic extent of sampling were fitted with R v4.2.1.

## Results

### Genetic diversity and effective population size

Expected heterozygosity (*H*_*E*_) ranged from 0.0022 (SHW) to 0.1072 (TMN) with an average of 0.0568 (Table 1), whereas observed heterozygosity (*H*_*O*_) ranged from low values in SHW (0.0019), MKN (0.0334), and YSO (0.0344) to somewhat higher (but still low) values in LTR (0.0627), TLC (0.0656), and TMN (0.0804; Table 1). The highest average weighted nucleotide diversity (*π*_*w*_) was found in population TMN (0.1061) while the lowest nucleotide diversity was found in SHW (0.0018) with an average of 0.0546 (Table 1). Similarly to average weighted nucleotide diversity, allelic richness (*Ar*) values were very low (Table 1). The highest allelic richness (*Ar*) was found in TMN (1.1060) while the lowest was found in SHW (1.0020) with an average of 1.0558 (Table 1). The inbreeding coefficient (*F*_*IS*_) was significantly positive in every population, except in SHW where the lower 95% CI was negative, indicating that nearly all of the populations were inbred (Table 1).

**Fig. 2:**
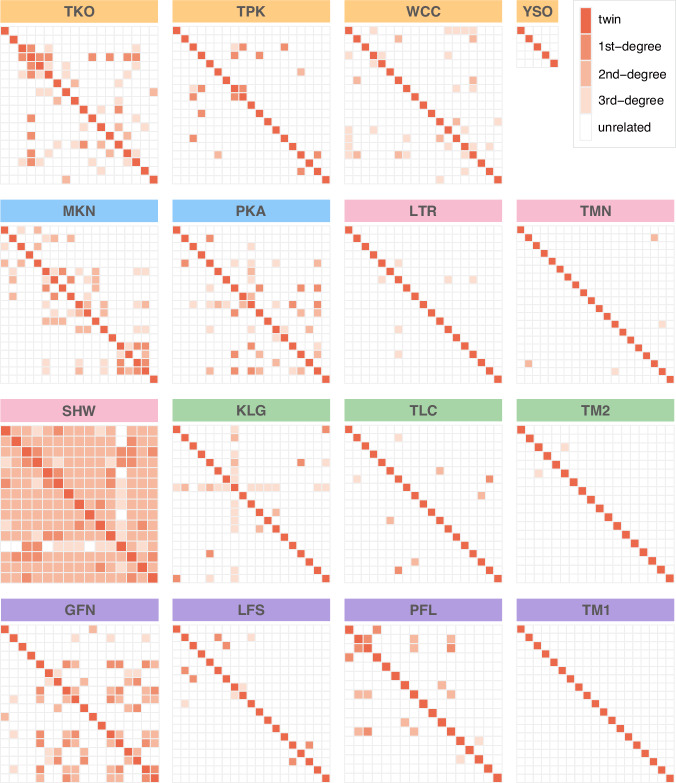
Relatedness between individuals within each of the 16 flat-headed loach populations. The different color boxes represent populations in different paleodrainage systems (see Fig. 1). Each row and column corresponds to a particular individual in a given population.

Kinship analyses further corroborated the results of genetic diversity analyses: there were significant differences in average relatedness among populations (ANOVA, *F*_*15,2423*_ = 136.7, *p* < 2e−16; Fig. 2; Supplementary Table S2 and Supplementary Fig. S1) and mean relatedness of individuals within each population correlated negatively with nucleotide diversity (Posterior mean = −0.293, *pMCMC* < 0.001) according to the mixed linear model (Supplementary Table S3). The SHW population had the highest mean relatedness with every individual being related to each other (and mostly sharing ~50% of their genetic information; Fig. 2), whereas all individuals in TM1 and YSO populations were unrelated (Fig. 2).

**Fig. 3:**
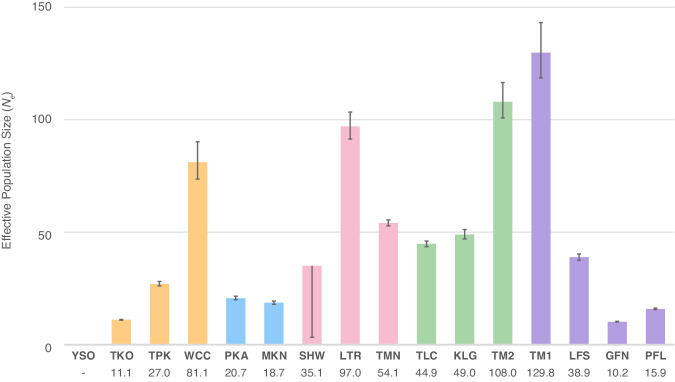
Estimates of contemporary effective population size (*N*_*e*_) in 16 loach populations as obtained with the program *NeEstimator* (Do et al. 2014). Vertical lines depict 95% Parametric CIs. CIs and *N*_*e*_ for SHW and YSO, respectively, were not estimable. The different colors represent populations in different paleodrainage systems (see Fig. 1).

The contemporary effective population size (*N*_*e*_) estimates were obtained for 15 populations ranging from 10.2 (GFN) to 129.8 (TM1) while estimation for YSO failed likely due to limited sampling size (*N*_*IND*_ = 5; Fig. 3). In addition, for SHW, *NeEstimator* failed to obtain the upper limit of 95% Parametric CI possibly because of the limited number of non-singleton loci (*N*_*ns*_ = 84) used in the estimation (Fig. 3 and Supplementary Table S4). A linear mixed-model was fitted to assess the effects of several variables on *N*_*e*_, which revealed that the altitude of the study site was positively associated with *N*_*e*_ (Posterior mean = 0.101, *pMCMC* = 0.042, see in Supplementary Table S3).

### Demographic history

**Fig. 4:**
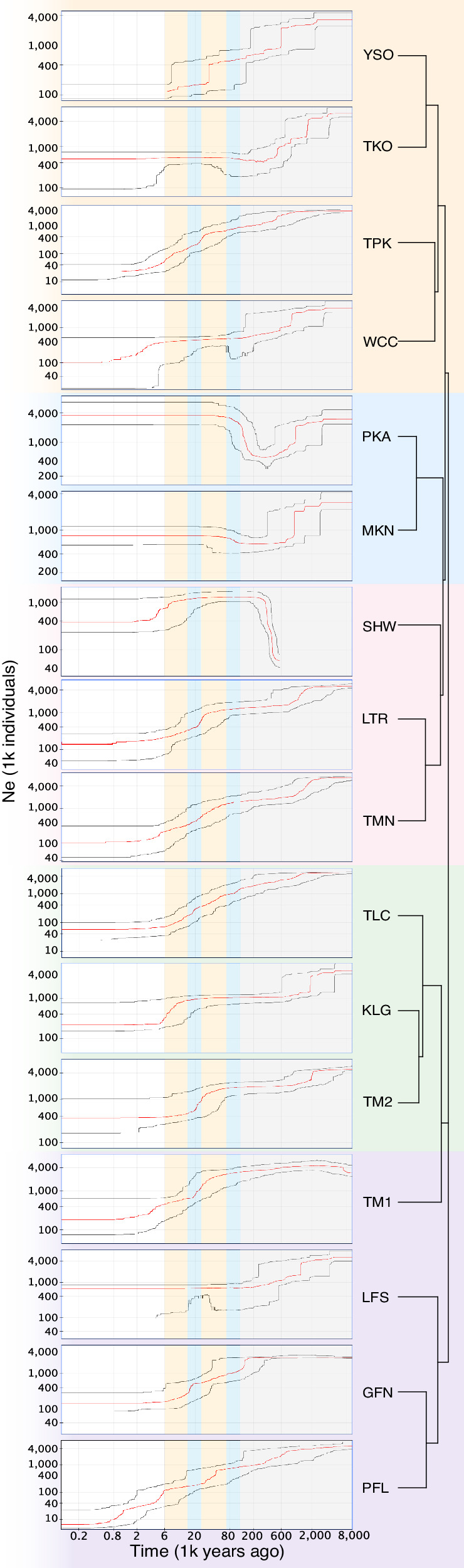
The demographic history of 16 flat-headed loach populations inferred by a folded SFS model projected to NJ tree. Different colors represent different paleodrainage systems (see Fig. 1). Red lines: the median estimated historical effective population sizes. Gray lines: 95% confidence interval of the inference. The periods of glaciation and interglaciation are highlighted with blue and orange vertical bars, respectively. The gray vertical area depicts a gap in the sedimentary record of Hong Kong.

We used a folded SNP frequency spectrum (SFS) model in the *Stairway Plot* to infer the demographic histories of the 16 populations. Almost every population (except SHW) appeared to have experienced at least one population size contraction before the first glaciation event inferred from the local sedimentary record (Fig. 4). During and after glaciation, only populations (PKA and MKN) at south Lantau drainage expanded after being bottlenecked while most (10 out of 16) of the populations continued to decline or appeared to have remained stable (Fig. 4).

### Genetic population structure

**Fig. 5:**
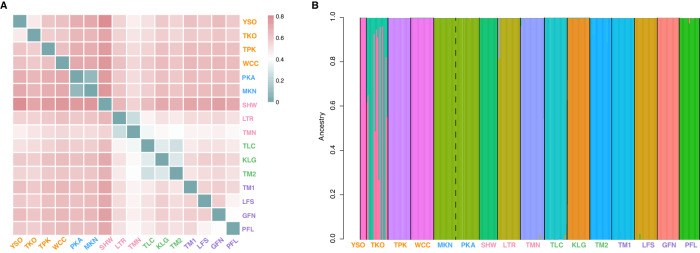
Population structure among sixteen flat-headed loach populations. The different font colors represent populations in different paleodrainage systems (see Fig. 1). **A** Pairwise *F*_*ST*_ estimates among populations. **B** Population structure inferred from admixture analysis assigning individuals to the optimal K = 16 (See Supplementary Fig. S3). Each colored bar represents one individual, and colored segments correspond to different ancestral components.

**Fig. 6:**
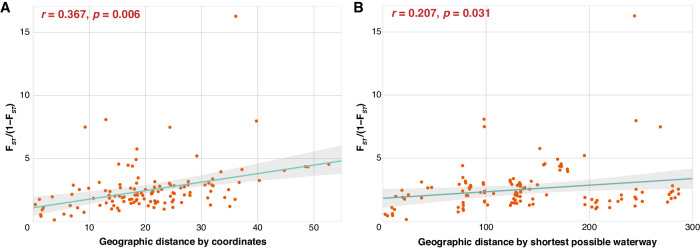
Isolation by distance across flat-headed loach populations. Isolation by distance with **A** geographic distance (km) calculated from site coordinates (*r* = 0.367, *p* = 0.006); and **B** from the shortest possible waterway based on the paleodrainage system map (*r* = 0.207, *p* = 0.031). Pairwise *F*_*ST*_ in both plots obtained with Rousset’s (1997) correction.

Pairwise *F*_*ST*_ among 16 populations ranged from 0.110 (MKN - PKA) to 0.942 (YSO - SHW; Fig. 5A, Supplementary Table S5) with an average of 0.668. All pairwise estimates were highly significant (*p* < 0.001; Supplementary Table S5). Population SHW showed the highest genetic differentiation from other populations, with an average *F*_*ST*_ of 0.825, followed by WCC with an average *F*_*ST*_ of 0.743. Population TMN exhibited the least genetic differentiation with an average *F*_*ST*_ of 0.372 to the other populations (Fig. 5A). There was nonetheless a clear, albeit relatively weak, pattern of isolation by distance (*p* < 0.05; Fig. 6). *t*-tests of *F*_*ST*_ within and among each paleodrainage system showed that populations originating from New Territories (NTW and NTE drainages) show equal level of differentiation within and among drainage systems while those from other drainages were more similar within the same drainage system (Supplementary Fig. S2). Admixture analyses corroborated the high *F*_*ST*_ values at K = 16 (Fig. 5B and Supplementary Fig. S3), none of the clusters exhibited evidence of among population admixture, except that PKA and MKN clustered together (Fig. 5B). Moreover, TKO consisted of two components, indicating that it could be a mixture of two ancestral populations (Fig. 5B).

Plotting the *F*_*ST*_ across the 27 studies (excluding the current study) against geographic extent of sampling shows that the degree of population differentiation increases as a function of geographic area sampled (Fig. 8; linear model: *F*_*1,25*_ = 4.538, *p* = 0.043). However, when the current study was included to the linear model, the relationship was no longer significant (*F*_*1,26*_ = 1.054, *p* = 0.314; See Supplementary Table S7 and Supplemental Material for details), indicating that the mean *F*_*ST*_ value of *O. platycephalus* was an outlier (Fig. 8).

**Fig. 7:**
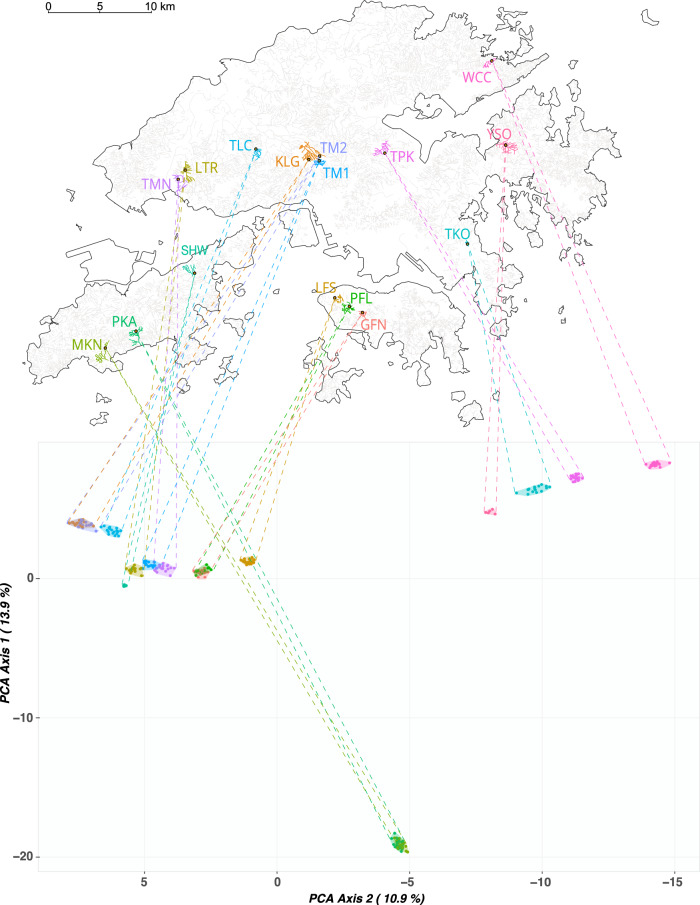
Results of principal component analysis (PCA) of allele frequencies projected onto the map of study area. Note that PCA Axis 2 is depicted on the x-axis.

Principal component analysis revealed the clearest distinction at Axis 1 between the two southern Lantau populations (PKA and MKN) and the rest of the populations (Fig. 7). Axis 2 further separated the East New Territories populations (TPK, TKO, WCC and YSO) from the rest (Fig. 7). There was also a clear pattern for populations from the same paleodrainage systems (Fig. 1) to cluster together: Tai Mo Shan system (TMS) - TM2, KLG and TLC; West New Territories system (NTW) - SHW, LTR and TMN; Hong Kong Island system (HKI) - PFL, GFN and LFS; South Lantau system (LTS) - PKA and MKN; East New Territories system (NTE) - TKO, TPK, WCC and YSO (Fig. 7; see Supplementary Fig. S4 for PCA plot excluding PKA and MKN populations). However, TM1 clustered with NTW populations although it was indicated to be part of the HKI drainage system (Fig. 1).

## Discussion

As we expected, *O. platycephalus* populations were genetically highly structured, low in genetic diversity and exhibited very low contemporary effective population sizes. Not surprisingly, all populations displayed positive inbreeding coefficients, and many showed signs of inbreeding as indicated by high relatedness estimators. Hence, all populations may be at risk of losing their adaptive potential, since *N*_*e*_ for populations to retain evolutionary potential is estimated to range from 500 to 1000 (Franklin and Frankham 1998; Frankham et al. 2014b), and estimates of less than 50 indicate that populations are in immediate danger (Hoban et al. 2020).

Low level of within-population genetic diversity is typical of freshwater fishes (DeWoody and Avise 2000; Martinez et al. 2018; Ward et al. 1994), and different measures of genetic diversity indicated low, but not exceptionally low genetic diversity within *O. platycephalus* populations. For instance, nucleotide diversities in isolated populations of three-spined stickleback (*Gasterosteus aculeatus*; Coll-Costa et al. 2024) and nine-spined sticklebacks (*Pungitius pungitius*; Kivikoski et al. 2023) are typically much lower than recorded for *O. platycephalus*. However, some caution is needed as different methods and filtering criteria have been used in different studies so genetic diversity estimates may not be strictly comparable (Korunes and Samuk 2021; Konopiński 2023). Nevertheless, loach average weighted nucleotide diversity (mean *π*_*w*_ = 0.0546) was slightly lower than that of an isolated and near-threatened wels catfish (*Silurus glanis; π* = 0.0690) population (Palm et al. 2019; Littmann 2022) and a highly genetically structured salmonid fish (*Salvelinus fontinalis;* π = 0.065; Ferchaud et al. 2020). Likewise, nucleotide diversity in *Achondrostoma salmantinum*, an endangered freshwater cyprinid endemic to Spain, was much higher (π range = 0.133–0.250; Corral-Lou et al. 2021) than in *O. platycephalus*. In the same vein, *Teleogramma*, a small clade of rheophilic cichlids from the Congo River showed similarly high levels of genetic diversity (mean π for each species ranging from 0.067 to 0.155; Alter et al. 2017). All this suggests comparably low levels of genetic diversity in *O. playtcephalus* and this inference is backed up by the extremely low estimates of loach effective population sizes. Direct comparisons to earlier genetic studies of Hong Kong freshwater fishes are difficult because they were based on mitochondrial DNA (Wong et al. 2017; Wong et al. 2019; Wu et al. 2019) which has ¼ of *N*_*e*_ of that of nuclear markers (Birky et al. 1983), or a handful of microsatellite markers (Wu et al. 2016) which have higher mutation rates than nuclear single nucleotide polymorphisms.

Effective population size is a fundamental parameter in the conservation context as its magnitude indicates the amount of genetic drift and inbreeding taking place (Charlesworth 2009). *O. platycephalus* populations with extremely low contemporary effective population sizes (cf. estimates in Palstra and Fraser 2012; Palstra and Ruzzante 2008) are thereby exposed to further erosion of genetic variability and greater risk of fixation of deleterious mutations. With contemporary effective population sizes below 50, it is likely that selection against deleterious mutations will be ineffective (Brandvain and Wright 2016; Lynch et al. 1995; Robinson et al. 2023) allowing even highly detrimental mutations to become fixed in loach populations. Unfortunately, there is no reference genome for *O. platycephalus* and due to the nature of the reduced representation approach used in this study, we cannot estimate the loads of detrimental mutations in the loach populations. Hence, an obvious and interesting question for follow-up studies would be to characterize the putative deleterious variation in these populations to assess the genomic consequences of prolonged population declines once genomic resources for this become available.

Another important consequence of low contemporary effective population size of *O. platycephalus* populations is their compromised ability to adapt to changing environmental conditions. Although our understanding of likelihood of adaptation to climate change is still limited (Merilä and Hendry 2014; Merilä and Lv 2024), low levels of genetic variability and low effective sizes of *O. platycephalus* populations suggest that they would have limited potential to adapt to sudden changes in conditions within their current range-limited habitats. In the context of a warming climate, these fishes are confined to their current habitats with no refuges to occupy further upstream. Interestingly, we found that populations at higher altitudes had larger contemporary effective population sizes than those from lower altitudes. This may be a reflection of the fact that the species is better adapted to lower-order upstream habitats of hillstreams and has been able to sustain larger effective sizes in these locations than in higher-order reaches further downstream. Such adaptation might be related to both abiotic (e.g. lower temperature) and biotic factors (e.g. a lack of competition with other fish species), but further studies would be needed to resolve this matter.

**Fig. 8:**
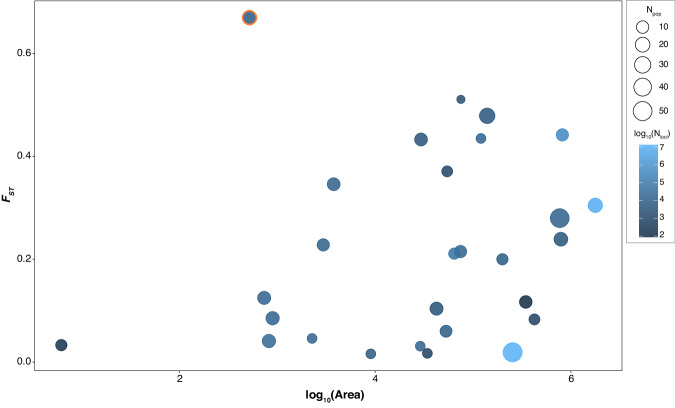
Bubble plot illustrating the relationship between *F*_*ST*_ and extent of geographic sampling area for studies of freshwater fishes published between 2013 and 2023 using single nucleotide polymorphism loci (SNPs). Bubble size indicates the number of populations and color indicates the number of SNP loci after log_10_ transformation. The *F*_*ST*_ estimate for loaches from the current study is highlighted with an orange circle. For details of the underlying data, see Supplementary Table S6 and Supplementary Material. For linear models fitted into this data, see Supplementary Table S7.

Given the clear footprint of strong genetic drift in *O. platycephalus* populations, it was not surprising that the degree of genetic differentiation among them was high with an average *F*_*ST*_ of 0.668. Although freshwater fish populations are typically more genetically structured than their marine counterparts (Ward et al. 1994), to the best of our knowledge, strong population structuring comparable to that observed in *O. platycephalus* has not been reported for any freshwater fish species from such a small geographic area (Fig. 8 and Supplementary Table S6). However, high levels of population differentiation have been reported among pond populations of nine-spined sticklebacks (*Pungitius pungitius*) which are characterized by strict isolation, with average *F*_*ST*_ = 0.49 (Shikano et al. 2010) comparable to - but slightly lower than - *O. platycephalus*. Similarly, two populations of a landlocked goby *Rhinogobius sp*. in Japan had an *F*_*ST*_ of 0.53 (Ohara et al. 2005). However, both these studies were based on microsatellite markers. We compiled data from published studies that used genome sequencing approaches and found that the degree of genetic differentiation among *O. platycephalus* populations was indeed exceptionally high given the limited geographic extent of sampling within Hong Kong.

Relatively high *F*_*ST*_ values have also been reported for two other Hong Kong loaches that inhabit larger streams than *O. plathycephalus*, although these estimates are not directly comparable as they were based on variation in mitochondrial control region (average *F*_*ST*_ = 0.66 in *Schistura fasciolata* and *F*_*ST*_ = 0.88 in *Pseudogastromyzon myersi*; Wong et al. 2017). Similarly, Wu et al. (2016) reported *F*_*ST*_ = 0.57 estimated using five microsatellite markers in the Hong Kong goby *Rhinogobius duospilus* indicating that, as has been noted for shrimps in the same habitats (Tsang et al. 2016; Yam and Dudgeon 2005), the limited connectivity of Hong Kong streams has contributed to high genetic divergence in their inhabitants.

Our reconstructions of historical demography revealed that nearly all *O. platycephalus* populations experienced population size declines before and during the last glaciation. Most but not all populations had also experienced continued population declines since then. These patterns accord with the likelihood that the local populations became isolated following the post-glacial sea level rise (Wong et al. 2017; Wu et al. 2016). The fact that some populations did not exhibit clear downward trends might be ascribable to methodological problems: the approaches used in historical demography reconstructions tend to lose resolution in modern times especially if sample sizes are low as in our study (Li and Durbin 2011; Liu and Fu 2015; Nadachowska‐Brzyska et al. 2022). Some support for this conjecture is provided by the observation that the three populations that did not show downward trends were among those with the smallest contemporary effective population sizes. Assuming an *N*_*e*_*/N*_*C*_ ratio of 10% (Frankham 1995; Hoban et al. 2020), our data suggests that contemporary population sizes (*N*_*C*_) of *O. platycephalus* range from ca. 100 to 1300 individuals. These are very small numbers when compared to many other species (cf. Palstra and Fraser 2012).

Given the findings of this study, what is the outlook for loach populations in Hong Kong hillstreams? Given that most populations have been subject to strong genetic drift and loss of genetic diversity, as well as the predicted further loss of diversity and increased risk of inbreeding depression in populations comprising fewer than 50 individuals (Franklin and Frankham 1998; Hoban et al. 2020), most (10/16) of the study populations can be expected undergo further loss of variability. Perhaps more seriously, none of the study populations appear to have an effective size large enough to permit adaptation to climate warming or other environmental changes: even the two populations with the highest contemporary *N*_*e*_ are far from the *N*_*e*_ = 500 mark considered necessary to preserve evolutionary potential, and much smaller than the more conservative *N*_*e*_ = 1000 benchmark (Frankham et al. 2014a). From this perspective, *O. platycephalus* populations in Hong Kong are at high risk of local extinctions, and may even represent an existing extinction debt.

It is interesting to speculate that extinctions have already taken place in hillstreams that we visited for this study where we were unable to find any loaches. Future studies might be able to address this possibility by analyzing environmental DNA from stream sediment cores (e.g. Nelson‐Chorney et al. 2019). Possible conservation measures for declining populations might also consider the reciprocal translocation of loaches between streams to enhance genetic diversity, reduce the negative consequences of inbreeding and enhance their adaptive potential in the face of future environmental changes. The two populations (MKN and PKA) in southern Lantau, which are the sole representatives of populations from the South Lantau drainage, should be prioritized for conservation due to their very small contemporary effective population sizes (≤20) and genetic distinctiveness from all other studied populations. Secondary priorities would be populations on Hong Kong Island (e.g. GFN and PFL) followed by those in the East New Territories (e.g. TKO and TPK).

In conclusion, the picture emerging from our analyses is that local flat-headed loach populations of Hong Kong became isolated after post-glacial sea level rise and have been subject to strong genetic drift in the subsequent absence of connectivity at the metapopulation level, i.e. a natural consequence of epoch rollover. As a consequence, the local populations have experienced loss of genetic diversity and increased genetic differentiation as manifested in extremely high *F*_*ST*_ estimates even over very short geographical distances. The low estimates of contemporary *N*_*e*_ suggest that local populations have severely compromised ability to remain viable with lowered evolutionary potential to meet the demands of a changing environment. Future studies based on whole genome resequencing data can refine this picture and give deeper insights into the levels (e.g. Robinson et al. 2019) and consequences (e.g. Keller and Waller 2002) of inbreeding in these populations. We further suggest that the *O. platycephalus* populations in Hong Kong can provide an excellent study system to document whether lowered genetic diversity is associated with increased extinction rate or not - a topic still controversial in conservation genetics (García-Dorado and Caballero 2021; DeWoody et al. 2021; Teixeira and Huber 2021; Willi et al. 2022).

### Supplementary information

Supporting information

## Data Availability

DArT-seq SNP genotyping datasets (SNPs report and vcf files) are openly available from Figshare (10.6084/m9.figshare.24792567).
