# Supplementary material for: Ecological genetics of isolated loach populations indicate compromised adaptive potential
Source: Heredity (Edinb). 2024 Jul 3;133(2):88–98. doi: 10.1038/s41437-024-00695-0 (PMC11286901; doi:10.1038/s41437-024-00695-0)
Supplement: Supplementary file 1 — Supporting information [file 41437_2024_695_MOESM1_ESM.docx]

**Supporting information for:**

**Ecological genetics of isolated loach populations indicate compromised adaptive potential**

Xi Wang, Kerry Reid, Ying Chen, David Dudgeon & Juha Merilä

Table of Contents:

| Table S1 | Page 2 |
| --- | --- |
| Table S2 | Page 3 |
| Table S3 | Page 3 |
| Table S4 | Page 4 |
| Table S5 | Page 4 |
| Table S6 | Page 5 |
| Table S7 | Page 7 |
| Figure S1 | Page 8 |
| Figure S2 | Page 9 |
| Figure S3 | Page 10 |
| Figure S4 | Page 11 |
| Supplemental Material | Page 12 |

Table S1. Information on flat-headed loach sampling sites

| Population | Abbreviation | Region | Paleodrainage system | Abbreviation of paleodrainage system | Latitude | Longitude |
| --- | --- | --- | --- | --- | --- | --- |
| Go Fu Nam Hang | GFN | Hong Kong Island | Hong Kong Island | HKI | 22.264 | 114.159 |
| Lung Fu Shan | LFS | Hong Kong Island | Hong Kong Island | HKI | 22.279 | 114.133 |
| Pok Fu Lam | PFL | Hong Kong Island | Hong Kong Island | HKI | 22.270 | 114.146 |
| Tai Mo Shan 1 | TM1 | New Territories | Hong Kong Island | HKI | 22.410 | 114.117 |
| Middle Kau Nga Hang | MKN | Lantau Island | South Lantau Island | LTS | 22.230 | 113.914 |
| Pak Kung Au | PKA | Lantau Island | South Lantau Island | LTS | 22.247 | 113.942 |
| Tseung Kwan O | TKO | New Territories | East New Territories | NTE | 22.330 | 114.259 |
| Tai Po Kau | TPK | New Territories | East New Territories | NTE | 22.416 | 114.181 |
| Wong Chuk Chung | WCC | New Territories | East New Territories | NTE | 22.504 | 114.283 |
| Yung Shue O | YSO | New Territories | East New Territories | NTE | 22.423 | 114.296 |
| Lam Tei Irrigation Reservoir | LTR | New Territories | West New Territories | NTW | 22.401 | 113.991 |
| Siu Ho Wan | SHW | Lantau Island | West New Territories | NTW | 22.303 | 113.999 |
| Tuen Mun | TMN | New Territories | West New Territories | NTW | 22.391 | 113.983 |
| Kap Lung | KLG | New Territories | Tai Mo Shan | TMS | 22.411 | 114.107 |
| Tai Lam Chung | TLC | New Territories | Tai Mo Shan | TMS | 22.419 | 114.059 |
| Tai Mo Shan 2 | TM2 | New Territories | Tai Mo Shan | TMS | 22.412 | 114.119 |

Table S2. Results of ANOVA of mean relatedness among 16 populations.

|  | *df* | *Sum Sq* | *Mean Sq* | *F* | *p* |
| --- | --- | --- | --- | --- | --- |
| Population | 15 | 14.39 | 0.959 | 136.7 | <2e-16*** |
| Residual | 2423 | 17.00 | 0.007 |  |  |

Table S3. Results of Bayesian linear model fitted with MCMC algorithm (Markov Chain Monte Carlo) assessing the effects of geographical factors (altitude, sum length of streams) and relatedness of individuals within each population on *π* and *N_e_*.

| Response variable | Explanatory variables | Posterior mean | pMCMC | 95% CI of Posterior mean |
| --- | --- | --- | --- | --- |
| *π_w_* | *N_e_* | 1.392e-4 | 0.452 | -2.707e-4 ~ 5.228e-4 |
|  | Relatedness | -0.293 | <0.001 *** | -0.452 ~ -0.128 |
|  | Altitude | -1.009e-5 | 0.793 | -9.193e-5 ~ 6.192e-5 |
|  | Sum Length of streams | 7.814e-7 | 0.579 | -2.599e-6 ~ 3.555e-6 |
| *N_e_* | *π_w_* | 4.066e+2 | 0.503 | -8.306e+2 ~ 1.646e+3 |
|  | Relatedness | -1.60 | 0.965 | -8.302e+2 ~ 7.508e+2 |
|  | Altitude | 0.101 | 0.042 * | 8.504e-4 ~ 0.193 |
|  | Sum Length of streams | 2.273e-3 | 0.317 | -2.566e-3 ~ 6.692e-3 |

Table S4. Estimates of current effective population size (*N_e_*) of flat-headed loaches.

| Population | *N_ns_* | *N_e_* | 95% Parametric low | 95% Parametric high |
| --- | --- | --- | --- | --- |
| GFN | 859 | 10.2 | 10.1 | 10.4 |
| KLG | 1564 | 49.0 | 47.0 | 51.2 |
| LFS | 1513 | 38.9 | 37.5 | 40.4 |
| LTR | 1904 | 97.0 | 91.4 | 103.4 |
| MKN | 888 | 18.7 | 18.0 | 19.4 |
| PFL | 1580 | 15.9 | 15.6 | 16.3 |
| PKA | 922 | 20.7 | 20.0 | 21.6 |
| SHW | 84 | 35.1 | 3.3 | - |
| TKO | 1531 | 11.1 | 10.9 | 11.3 |
| TLC | 2113 | 44.9 | 43.6 | 46.1 |
| TM1 | 1630 | 129.8 | 118.6 | 143.2 |
| TM2 | 1925 | 108.0 | 100.8 | 116.5 |
| TMN | 2536 | 54.1 | 52.8 | 55.5 |
| TPK | 1218 | 27.0 | 26.1 | 28.1 |
| WCC | 1117 | 81.1 | 73.6 | 90.2 |
| YSO | 710 | - | - | - |

*N_ns_*: Number of non-singleton loci used for estimating *N_e_*; “-”: No estimate available or generated by the program.

Table S5. Pairwise *F_ST_* among 16 flat-headed loach populations.

| Pop | YSO | TKO | TPK | WCC | PKA | MKN | SHW | LTR | TMN | TLC | KLG | TM2 | TM1 | LFS | GFN | PFL |
| --- | --- | --- | --- | --- | --- | --- | --- | --- | --- | --- | --- | --- | --- | --- | --- | --- |
| YSO |  | 0.000* | 0.000* | 0.000* | 0.000* | 0.000* | 0.000* | 0.000* | 0.000* | 0.000* | 0.000* | 0.000* | 0.000* | 0.000* | 0.000* | 0.000* |
| TKO | 0.562 |  | 0.000* | 0.000* | 0.000* | 0.000* | 0.000* | 0.000* | 0.000* | 0.000* | 0.000* | 0.000* | 0.000* | 0.000* | 0.000* | 0.000* |
| TPK | 0.710 | 0.675 |  | 0.000* | 0.000* | 0.000* | 0.000* | 0.000* | 0.000* | 0.000* | 0.000* | 0.000* | 0.000* | 0.000* | 0.000* | 0.000* |
| WCC | 0.725 | 0.687 | 0.728 |  | 0.000* | 0.000* | 0.000* | 0.000* | 0.000* | 0.000* | 0.000* | 0.000* | 0.000* | 0.000* | 0.000* | 0.000* |
| PKA | 0.802 | 0.756 | 0.798 | 0.813 |  | 0.000* | 0.000* | 0.000* | 0.000* | 0.000* | 0.000* | 0.000* | 0.000* | 0.000* | 0.000* | 0.000* |
| MKN | 0.813 | 0.765 | 0.805 | 0.819 | 0.110 |  | 0.000* | 0.000* | 0.000* | 0.000* | 0.000* | 0.000* | 0.000* | 0.000* | 0.000* | 0.000* |
| SHW | 0.942 | 0.839 | 0.882 | 0.888 | 0.882 | 0.890 |  | 0.000* | 0.000* | 0.000* | 0.000* | 0.000* | 0.000* | 0.000* | 0.000* | 0.000* |
| LTR | 0.656 | 0.652 | 0.696 | 0.716 | 0.693 | 0.703 | 0.756 |  | 0.000* | 0.000* | 0.000* | 0.000* | 0.000* | 0.000* | 0.000* | 0.000* |
| TMN | 0.544 | 0.566 | 0.610 | 0.637 | 0.603 | 0.614 | 0.648 | 0.296 |  | 0.000* | 0.000* | 0.000* | 0.000* | 0.000* | 0.000* | 0.000* |
| TLC | 0.601 | 0.610 | 0.649 | 0.679 | 0.667 | 0.675 | 0.745 | 0.521 | 0.421 |  | 0.000* | 0.000* | 0.000* | 0.000* | 0.000* | 0.000* |
| KLG | 0.695 | 0.675 | 0.717 | 0.737 | 0.734 | 0.743 | 0.815 | 0.595 | 0.499 | 0.370 |  | 0.000* | 0.000* | 0.000* | 0.000* | 0.000* |
| TM2 | 0.642 | 0.636 | 0.683 | 0.704 | 0.697 | 0.706 | 0.776 | 0.557 | 0.461 | 0.306 | 0.351 |  | 0.000* | 0.000* | 0.000* | 0.000* |
| TM1 | 0.717 | 0.694 | 0.742 | 0.760 | 0.745 | 0.753 | 0.831 | 0.616 | 0.515 | 0.559 | 0.637 | 0.573 |  | 0.000* | 0.000* | 0.000* |
| LFS | 0.689 | 0.669 | 0.726 | 0.744 | 0.731 | 0.739 | 0.820 | 0.621 | 0.524 | 0.585 | 0.660 | 0.613 | 0.654 |  | 0.000* | 0.000* |
| GFN | 0.744 | 0.704 | 0.753 | 0.771 | 0.752 | 0.760 | 0.852 | 0.632 | 0.528 | 0.591 | 0.676 | 0.624 | 0.676 | 0.662 |  | 0.000* |
| PFL | 0.695 | 0.670 | 0.720 | 0.739 | 0.718 | 0.726 | 0.817 | 0.595 | 0.495 | 0.555 | 0.639 | 0.589 | 0.635 | 0.519 | 0.479 |  |

The value below the diagonal is the pairwise *F_ST_* and the value about the diagonal is the *p*-value, *indicates a significant difference after Bonferroni correction (*p* < 0.001).

Table S6. The mean pairwise *F_ST_* values of other freshwater fish species obtained using reduced-representation genome sequencing published between 2013 and 2023. For references, see Supplementary materials.

| Species | *N_pop_* | *N_ind_* | *N_loci_* | Geographic Area | Sampling area (km^2^) | Mean *F_ST_* | References |
| --- | --- | --- | --- | --- | --- | --- | --- |
| *Barbatula barbatula* | 14 | 255 | 17407 | Belgium | 896.61 | 0.085 | Deflem et al., 2022 |
| *Cyprinus carpio* | 4 | 106 | 17828 | Southern Caspian Basin | 2267.80 | 0.046 | Jafari et al., 2022 |
| *Esox lucius* | 16 | 351 | 4329 | Alaska | 789136.79 | 0.239 | Campbell et al., 2023 |
|  | 4 | 121 | 14124 | Xinjiang, China | 28885.91 | 0.031 | Luan et al., 2021 |
| *Etheostoma caeruleum* | 10 | 98 | 13820 | central North America | 2952.40 | 0.228 | Luiken et al., 2021 |
| *Gasterosteus aculeatus* | 13 | 236 | 17411 | Belgium | 734.95 | 0.125 | Deflem et al., 2022 |
| *Labeobarbus natalensis* | 4 | 21 | 723 | KwaZulu-Natal Province, South Africa | 34155.14 | 0.017 | Stobie et al., 2018 |
| *Monopterus albus* | 19 | 137 | 8941794 | China | 1773167.37 | 0.305 | Lv et al., 2022 |
| *Morone saxatilis* | 9 | 438 | 9352 | North America | 52945.98 | 0.060 | Wojtusik et al., 2023 |
| *Nannoperca australis* | 25 | 263 | 5162 | Murray–Darling Basin, Australia | 139318.03 | 0.479 | Brauer et al., 2016 |
| *Oreochromis niloticus* | 13 | 171 | 2950 | Benin | 42362.22 | 0.104 | Fagbémi et al., 2021 |
| *Pangasianodon hypophthalmus* | 7 | 93 | 7263 | Mekong river, Southeast Asia | 199384.52 | 0.200 | Vu et al., 2020 |
| *Pseudorasbora parva* | 14 | 249 | 23401 | Belgium | 822.60 | 0.041 | Deflem et al., 2022 |
| *Rhamdia quelen* | 10 | 74 | 17559 | Uruguay | 74488.75 | 0.215 | Ríos et al., 2020 |
| *Rhinichthys osculus* | 10 | 175 | 421929 | California, USA | 816471.39 | 0.442 | Su et al., 2022 |
| *Salmo trutta* | 7 | 167 | 182 | Taurion River, France | 6.23 | 0.033 | Saint-Pe et al., 2019 |
|  | 11 | 256 | 94 | UK & France | 345565.71 | 0.117 | Osmond et al., 2023 |
| *Salvelinus alpinus* | 12 | 369 | 14187 | Iceland | 3770.72 | 0.346 | Brachmann et al., 2021 |
|  | 50 | 1416 | 14779 | Canada | 767561.96 | 0.280 | Ferchaud et al., 2020 |
| *Salvelinus namaycush* | 12 | 107 | 3925 | Laurentian Shield lakes, Canada | 29419.18 | 0.433 | Bernatchez et al., 2016 |
|  | 4 | 486 | 6822 | Lake Superior, Canada | 9027.89 | 0.016 | Perreault-Payette et al., 2017 |
| *Sander vitreus* | 4 | 93 | 14529 | USA | 120381.07 | 0.435 | Johnson et al., 2023 |
|  | 3 | 60 | 2782 | USA | 75141.37 | 0.511 | Zhao et al., 2020 |
|  | 6 | 1069 | 796 | Great Lakes | 423602.62 | 0.083 | Euclide et al., 2022 |
|  | 6 | 120 | 1081 | Canada | 54402.13 | 0.371 | Lucchetti et al., 2018 |
| *Saurogobio dabryi* | 7 | 120 | 58930 | Yangtze River, China | 64315.65 | 0.211 | Liu et al., 2022 |
| *Oncorhynchus tshawytscha* | 53 | 3566 | 12996534 | USA | 253561.81 | 0.019 | Horn & Narum, 2023 |
| *Oreonectes platycephalus* | 16 | 282 | 7045 | Hong Kong | 520.9 | 0.670 | This study |

*N_pop_*: Number of populations; *N_ind_*: Number of individuals; *N_loci_*: Number of SNPs. Sampling area was estimated as the area of a polygon delimiting the geographic distribution of sampling sites.

Table S7. Results of linear models assessing *F_ST_* as a function of geographic area of sampling.

| Coefficients | Estimate | SE | R^2^ | Adjusted R^2^ | *F* | *df* | *t* | *p* |
| --- | --- | --- | --- | --- | --- | --- | --- | --- |
| Log_10_(Area) (27 studies of other freshwater fish species) | 0.052 | 0.024 | 0.154 | 0.120 | 4.538 | 25 | 2.130 | 0.043* |
| Log_10_(Area) (this study + 27 studies of other freshwater fish species) | 0.029 | 0.028 | 0.039 | 0.002 | 1.054 | 26 | 1.027 | 0.314 |


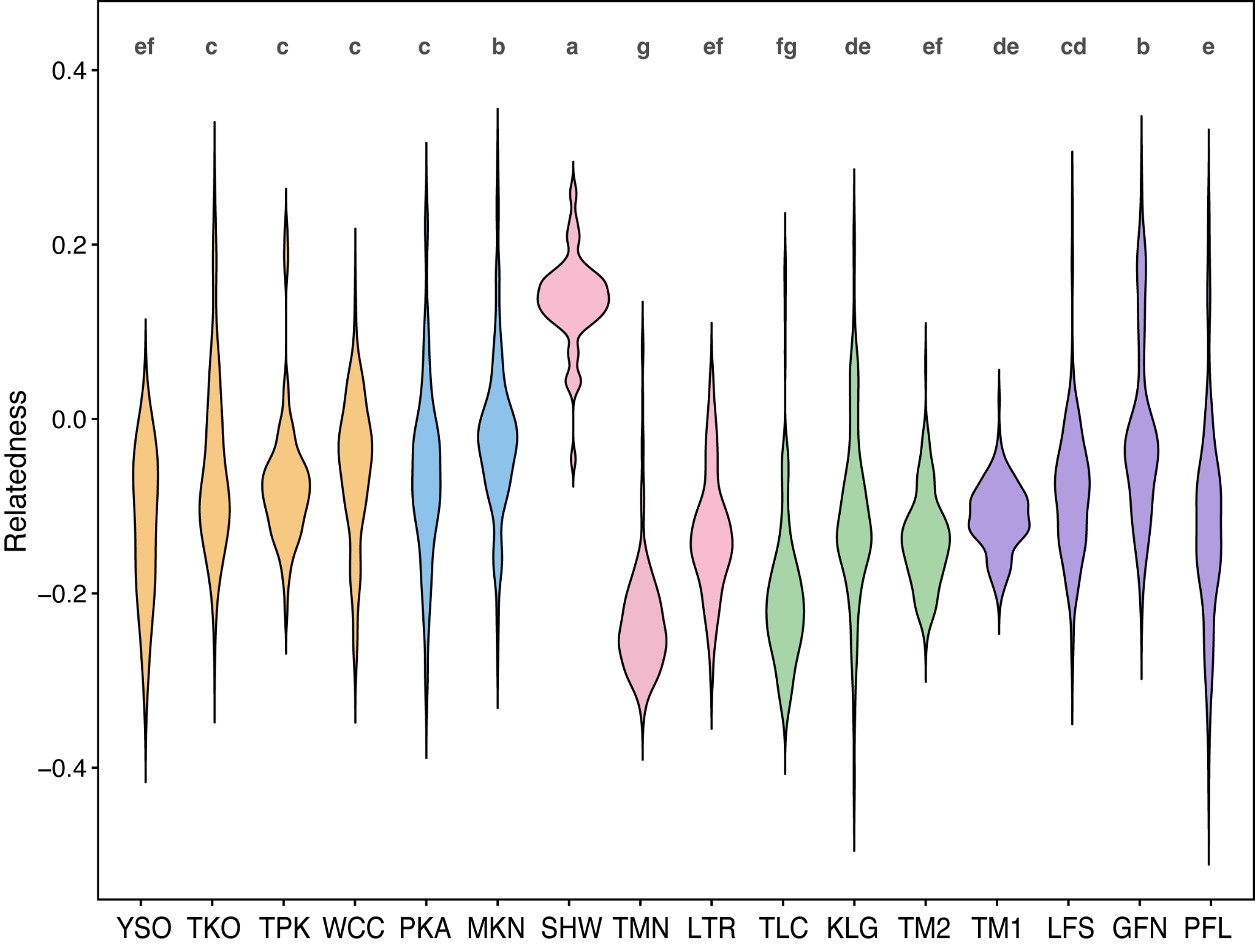


Figure S1. Mean relatedness among 16 flat-headed loach populations. Populations marked with different alphabets on the top indicate significant differences following FDR correction (*p* < 0.05). The different color violin represents populations in different paleodrainage systems (see Fig. 1).


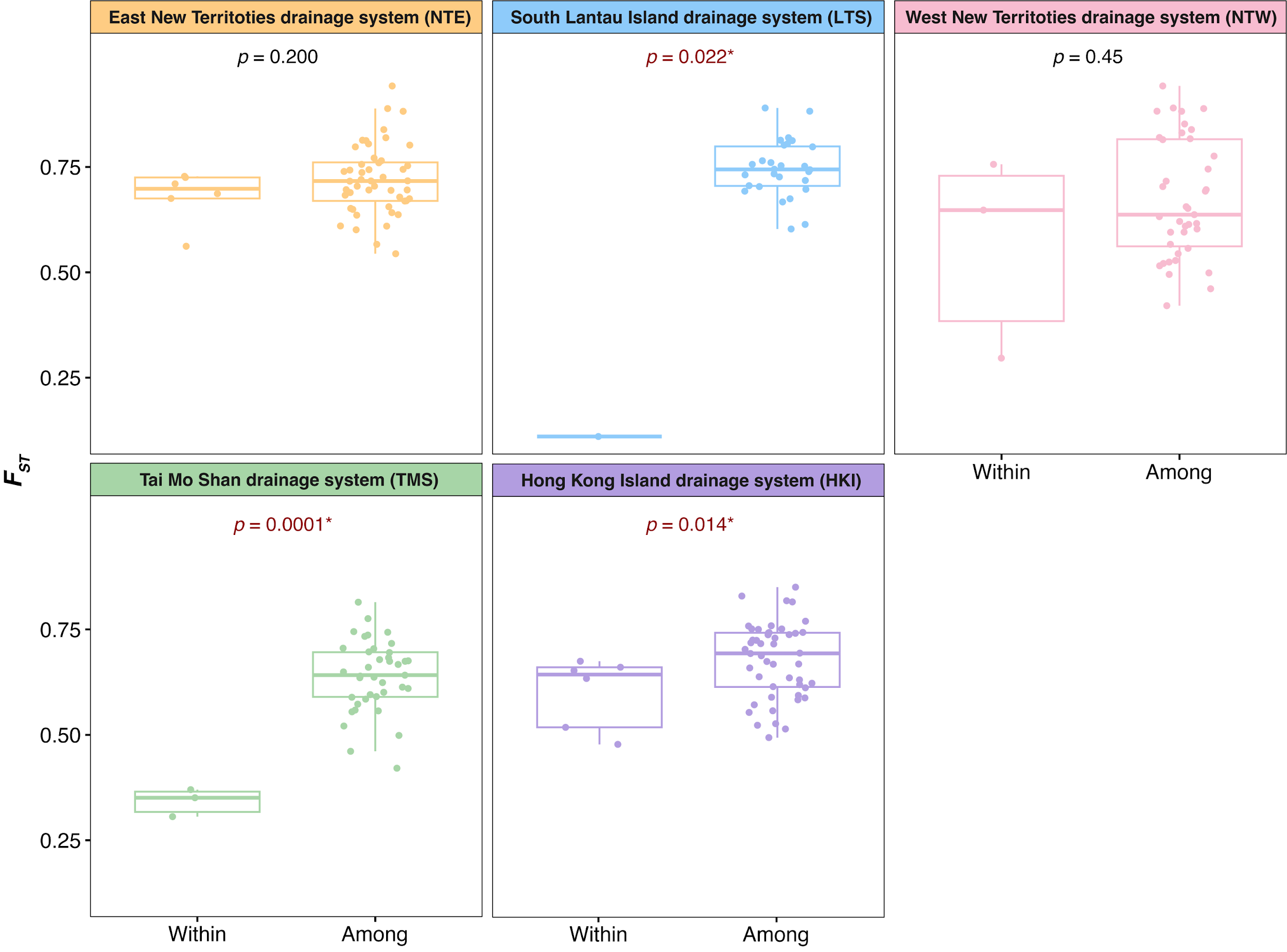


Figure S2. Comparison of *F_ST_* within and among each paleodrainage system. The *p*-values are from t-tests comparing *F_ST_* estimates within and among paleodrainage systems. *indicates significant difference between two *F_ST_* values.


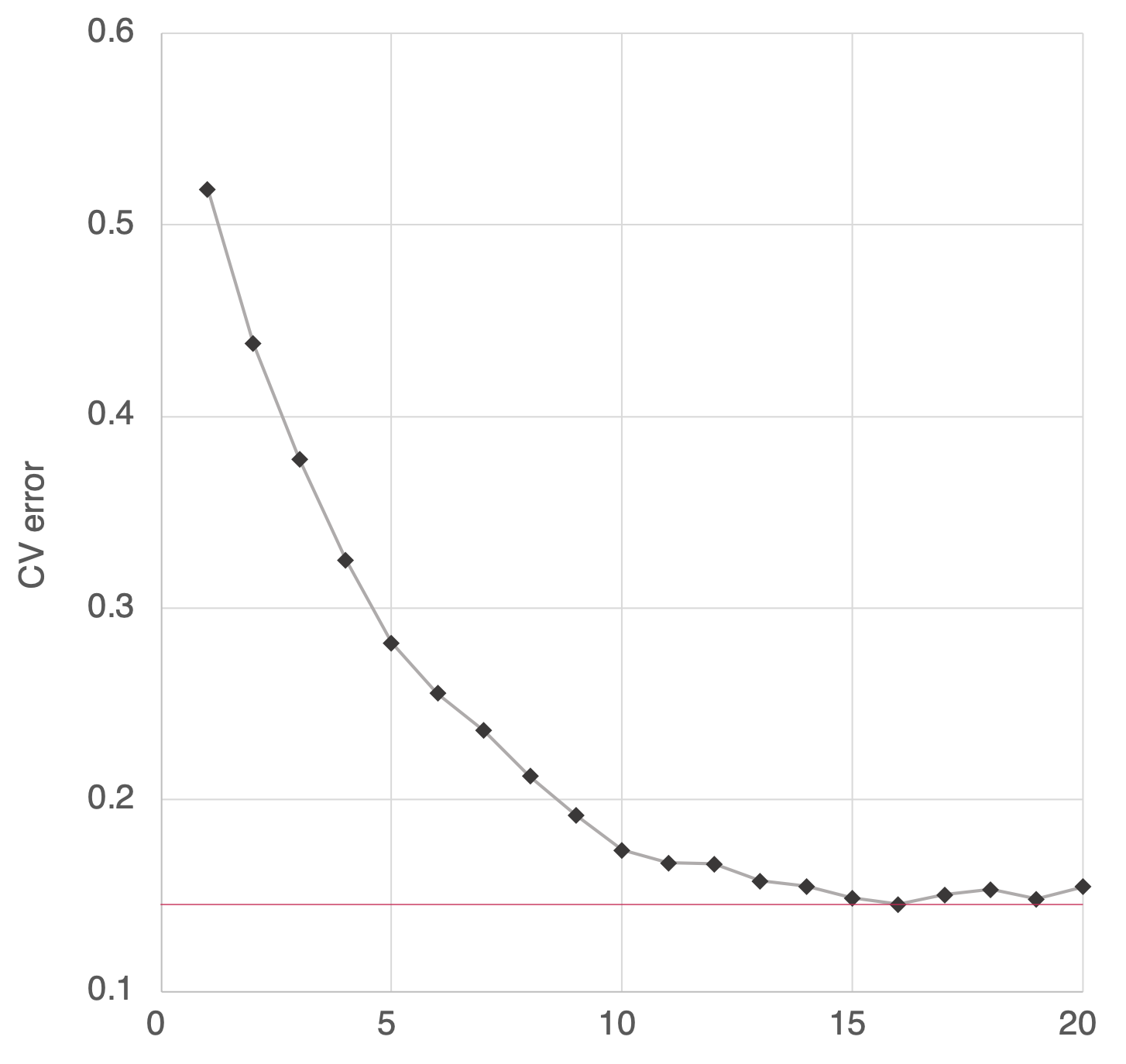


Figure S3. Plot of admixture Cross Validation (CV) error for K = 1 to 16 in sixteen *O. platycephalus* populations. K = 16 is best for admixture analysis as it minimizes the error.


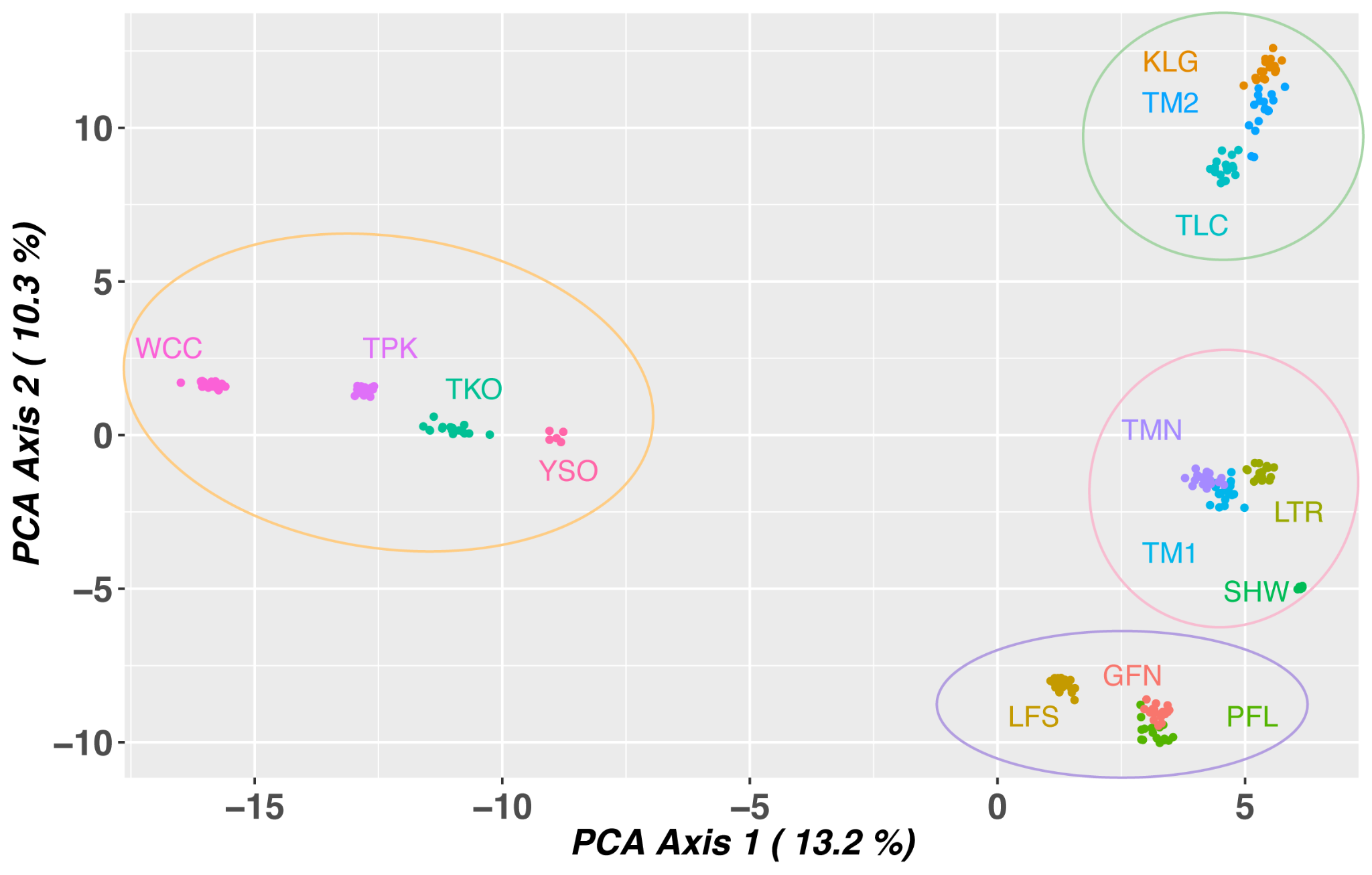


Figure S4. Principal component analysis (PCA) of *O. platycephalus* samples conducted excluding populations MKN and PKA.

**Supplemental Material**

***SNPs filtering***

Steps “ (i)” to “(vi)” are basic filterings for DArT dataset (<http://georges.biomatix.org/storage/app/media/Tutorial_5_dartR_Basic_Filtering.pdf>).

(i) reproducibility < 0.99 (default setting);

SNP datasets generated by DArT have an index, RepAvg, generated by reproducing the data independently for 30 of alleles that give a repeatable result, averaged over both alleles for each locus. repeatability is the percentage of scores that are repeated in the technical replicate dataset.

(ii) coverage < 5X or > 50X (default setting, coverage automatically calculated by dartR);

SNP datasets generated by DArT report AvgCountRef and AvgCountSnp as counts of sequence tags for the reference and alternate alleles respectively. These can be used to back calculate Read Depth.

(iii) SNPs with sequence tag length < 20 or > 69 (default setting);

SNP datasets generated by DArT typically have sequence tag lengths ranging from 20 to 69 base pairs.

(iv) SNP position outside the trimmed sequence tag (default setting);

Filter those the SNP position is outside the trimmed sequence tag.

(v) secondary SNPs (default setting);

SNP datasets generated by DArT include fragments with more than one SNP and record them separately with the same CloneID. These multiple SNP loci within a fragment (secondaries) are likely to be linked so should be filtered out all but the first sequence tag with the same CloneID.

(vi) pairwise Hamming distance between sequence tags < 0.2 (default setting);

Filter out loci that differ from each other by less than a specified number of base pairs.

(vii) call rate < 0.7; (viii) minor allele count (MAC) < 3.

The purpose of filtering call rate is to limit the proportion of missing data which would affect downstream analysis and the purpose of filtering for MAC is to remove possible sequencing errors.

***F_ST_ comparison***

To yardstick the level of population differentiation in *O. platycephalus*, we conducted a literature search for studies which had estimated the degree of population differentiation in other freshwater fishes using single nucleotide polymorphisms (SNPs) to estimate *F_ST_*. Using *Web of Science* we search publication with "fish (Topic) AND pairwise F*_ST_* (Topic) AND freshwater (Topic) OR lake (Topic) OR river (Topic) AND SNP (Topic)" and limited the search to last five years in twelve journals - *Aquatic Conservation Marine and Freshwater Ecosystems*; *Aquatic Ecology*; *Canadian Journal of Fisheries and Aquatic Sciences*; *Ecology*; *Ecology and Evolution*; *Ecological Applications*; *Freshwater Biology*; *Frontiers in Ecology and Evolution*; *Journal of Fish Biology*; *Journal of Freshwater Ecology*; *Molecular Ecology*; *Nature Ecology & Evolution* (1478 results). Using *Google Scholar* we searched publications with terms "fish; freshwater; *F_ST_*; genetic differentiation; RAD-Seq; SNP; wild" and limited the search to years 2013-2023 (452 results). Only published studies with completed peer-review were included (87 results).

The search results were further manually filtered based on the following criteria: 1) the study object should be freshwater fish species; 2) the study is based on wild rather than aquaculture material; 3) the study should use single nucleotide polymorphisms (SNPs) to estimate *F_ST_* . After pruning, 27 studies were retained to be included in the *F_ST_* comparison (Table S6). From these studies, we extracted the *F_ST_* , number of populations included, number of individuals used, number of loci used and estimated the extent of geographic area of sampling based on information provided in each of the papers. We conducted a series of linear models modeling *F_ST_* as function extracted explanatory variables (number populations, number of individuals, number of loci, geographic area of sampling) and found that the best model included only the geographic area as the explanatory variable (Table S7). However, after inclusion of the current study into the model, the geographic area of sampling was no longer significant (Table S7).

***References***

Allen, B. E., Bowles, E., Morris, M. R. J., & Rogers, S. M. (2017). Loss of SNP genetic diversity following population collapse in a recreational walleye (*Sander vitreus*) fishery. *Canadian Journal of Fisheries and Aquatic Sciences*, *75*(10), 1644-1651.

Bernatchez, S., Laporte, M., Perrier, C., Sirois, P., & Bernatchez, L. (2016). Investigating genomic and phenotypic parallelism between piscivorous and planktivorous lake trout (*Salvelinus namaycush*) ecotypes by means of RADseq and morphometrics analyses. *Molecular Ecology*, *25*(19), 4773-4792.

Brachmann, M. K., Parsons, K., Skúlason, S., & Ferguson, M. M. (2021). The interaction of resource use and gene flow on the phenotypic divergence of benthic and pelagic morphs of Icelandic Arctic charr (*Salvelinus alpinus*). *Ecology and Evolution*, *11*(12), 7315-7334.

Brauer, C. J., Hammer, M. P., & Beheregaray, L. B. (2016). Riverscape genomics of a threatened fish across a hydroclimatically heterogeneous river basin. *Molecular Ecology*, *25*(20), 5093-5113.

Campbell, M. A., Hale, M. C., Jalbert, C. S., Dunker, K., Sepulveda, A. J., López, J. A., Falke, J. A., & Westley, P. A. H. (2023). Genomics reveal the origins and current structure of a genetically depauperate freshwater species in its introduced Alaskan range. *Evolutionary Applications*, *16*(6), 1119-1134.

Deflem, I. S., Calboli, F. C. F., Christiansen, H., Hellemans, B., Raeymaekers, J. A. M., & Volckaert, F. A. M. (2022). Contrasting population genetic responses to migration barriers in two native and an invasive freshwater fish. *Evolutionary Applications*, *15*(12), 2010-2027.

Euclide, P. T., Larson, W. A., Bootsma, M., Miller, L. M., Scribner, K. T., Stott, W., Wilson, C. C., & Latch, E. K. (2022). A new GTSeq resource to facilitate multijurisdictional research and management of walleye *Sander vitreus*. *Ecology and Evolution*, *12*(12), e9591.

Fagbémi, M. N. A., Pigneur, L.-M., André, A., Smitz, N., Gennotte, V., Michaux, J. R., Mélard, C., Lalèyè, P. A., & Rougeot, C. (2021). Genetic structure of wild and farmed Nile tilapia (*Oreochromis niloticus*) populations in Benin based on genome wide SNP technology. *Aquaculture*, *535*, 736432.

Ferchaud, A.-L., Leitwein, M., Laporte, M., Boivin-Delisle, D., Bougas, B., Hernandez, C., Normandeau, É., Thibault, I., & Bernatchez, L. (2020). Adaptive and maladaptive genetic diversity in small populations: Insights from the brook charr (*Salvelinus fontinalis*) case study. *Molecular Ecology*, *29*(18), 3429-3445.

Horn, R. L., & Narum, S. R. (2023). Genomic variation across Chinook salmon populations reveals effects of a duplication on migration alleles and supports fine scale structure. *Molecular Ecology*, *32*(11), 2818-2834.

Jafari, O., Zeinalabedini, M., Robledo, D., Fernandes, J. M. O., Hedayati, A.-A., & Arefnezhad, B. (2022). Genotyping-by-Sequencing Reveals the Impact of Restocking on Wild Common Carp Populations of the Southern Caspian Basin. *Frontiers in Ecology and Evolution*, *10*, 872176.

Johnson, A., Zipfel, K., Hallerman, E., Massure, W., Euclide, P., & Welsh, A. (2023). Genomic evaluation of native walleye in the Appalachian region and the effects of stocking. *Transactions of the American Fisheries Society*, *152*(3), 346-360.

Liu, H., Xiong, F., Zhai, D., Duan, X., Chen, D., Chen, Y., Wang, Y., & Xia, M. (2022). Genetic Diversity and Population Differentiation of Chinese Lizard Gudgeon (*Saurogobio dabryi*) in the Upper Yangtze River. *Frontiers in Ecology and Evolution*, *10*, 890475.

Luan, P., Huo, T., Ma, B., Song, D., Zhang, X., & Hu, G. (2021). Genomic inbreeding and population structure of northern pike (*Esox lucius*) in Xinjiang, China. *Ecology and Evolution*, *11*(10), 5657-5668.

Luiken, J. M., Gamble, T., & Berendzen, P. B. (2021). Comparative riverscape genomics of the rainbow darter (*Etheostoma caeruleum*) in glaciated and unglaciated environments. *Ecology and Evolution*, *11*(24), 18305-18318.

Lv, W., Yuan, Q., Huang, W., Sun, X., Lv, W., & Zhou, W. (2022). Asian Swamp eel Monopterus albus Population Structure and Genetic Diversity in China. *Frontiers in Genetics*, *13*, 898958.

Osmond, D. R., King, R. A., Stockley, B., Launey, S., & Stevens, J. R. (2023). A low-density single nucleotide polymorphism panel for brown trout (*Salmo trutta* L.) suitable for exploring genetic diversity at a range of spatial scales. *Journal of Fish Biology*, *102*(1), 258-270.

Perreault-Payette, A., Muir, A. M., Goetz, F., Perrier, C., Normandeau, E., Sirois, P., & Bernatchez, L. (2017). Investigating the extent of parallelism in morphological and genomic divergence among lake trout ecotypes in Lake Superior. *Molecular Ecology*, *26*(6), 1477-1497.

Ríos, N., Casanova, A., Hermida, M., Pardo, B. G., Martínez, P., Bouza, C., & García, G. (2020). Population genomics in *Rhamdia quelen* (Heptapteridae, Siluriformes) reveals deep divergence and adaptation in the neotropical region. *Genes*, *11*(1), 109.

Saint-Pé, K., Leitwein, M., Tissot, L., Poulet, N., Guinand, B., Berrebi, P., Marselli, G., Lascaux, J.-M., Gagnaire, P.-A., & Blanchet, S. (2019). Development of a large SNPs resource and a low-density SNP array for brown trout (*Salmo trutta*) population genetics. *BMC Genomics*, *20*(1), 582.

Stobie, C. S., Oosthuizen, C. J., Cunningham, M. J., & Bloomer, P. (2018). Exploring the phylogeography of a hexaploid freshwater fish by RAD sequencing. *Ecology and Evolution*, *8*(4), 2326-2342.

Su, Y., Moyle, P. B., Campbell, M. A., Finger, A. J., O'Rourke, S. M., Baumsteiger, J., & Miller, M. R. (2022). Population genomic analysis of the speckled dace species complex identifies three distinct lineages in California. *Transactions of the American Fisheries Society*, *151*(6), 695-710.

Vu, N. T., Ha, T. T. T., Thuy, V. T. B., Trang, V. T., & Nguyen, N. H. (2020). Population genomic analyses of wild and farmed striped catfish *Pangasianodon hypophthalmus* in the lower Mekong River. *Journal of Marine Science and Engineering*, *8*(6), 471.

Wojtusik, K. J., Berlinsky, D. L., Kenter, L. W., & Kovach, A. I. (2023). River-of-origin assignment of migratory striped bass, with implications for mixed-stock analysis. *Transactions of the American Fisheries Society*, *152*(1), 15-34.

Zhao, H., Silliman, K., Lewis, M., Johnson, S., Kratina, G., Rider, S. J., Stepien, C. A., Hallerman, E. M., Beck, B., Fuller, A., & Peatman, E. (2020). SNP analyses highlight a unique, imperiled southern walleye (*Sander vitreus*) in the Mobile River Basin. *Canadian Journal of Fisheries and Aquatic Sciences*, *77*(8), 1366-1378.
